# Adverse Childhood Experiences: The Protective and Therapeutic Potential of Nature

**DOI:** 10.3389/fpsyg.2020.597935

**Published:** 2020-11-26

**Authors:** Anna K. Touloumakos, Alexia Barrable

**Affiliations:** ^1^Department of Psychology, Panteion University, Athens, Greece; ^2^Centre on Skills, Knowledge and Organisational Performance, Department of Education, University of Oxford, Oxford, United Kingdom; ^3^Department of Education Sciences, Hellenic Open University, Patras, Greece; ^4^School of Education and Social Work, University of Dundee, Dundee, United Kingdom

**Keywords:** adverse childhood experiences, trauma, psychopathology, nature, nature-based therapies, nature-based interventions, protective factors

## Abstract

Adverse childhood experiences (ACEs) are prevalent in many western populations. Large studies have put the likelihood of having at least one ACE above 50% of the general population. ACEs and the associated experience of chronic stress, moreover, have been consistently linked with a variety of negative physical and psychological health outcomes across the lifespan from behavioral problems and cognitive difficulties early on, to greater chance of suffering from a mental health disorder and engaging in self destructing behaviors. The literature puts forward several protective factors, such as mother-child relations, parental health, and community engagement. In this perspective paper we put forward the potential of regular nature engagement as a possible additional protective factor. Nature’s therapeutic potential has been well documented, for many psychopathologies and mental health difficulties. Yet studies looking at the protective and therapeutic potential of nature with people with ACEs are remarkably limited in numbers. In this perspective piece we conduct a search of the literature to find previous applications of nature as a protective or therapeutic intervention for people with ACEs. We highlight the gap in the current literature, and put forward various mechanisms of action that justify a closer exploration of this area in further research.

## Introduction

The term adverse and traumatic experiences was first used in the adverse childhood experiences (ACE) study conducted in the Kaiser Permanente Centers for Disease Control and Prevention back in the 1990s. ACEs include family abuse (physical, sexual, emotional), neglect (physical and emotional), and family dysfunction (domestic violence, substance abuse, mental illness, separation/divorce, imprisonment of family member) (Felitti et al., 1998). The term has been widely used since to refer to these 10 experiences, although additional adverse experiences have been acknowledged in the literature, such as for example economic adversity (see McLaughlin et al., 2012). ACEs have been acknowledged as an important public health issue: a recent large-scale United States study suggests that as many as the 61.55% of the participants report at least one ACE and 24.64% of the participants report three or more ACEs (Merrick et al., 2018). The unfavorable and pervasive impact of ACEs across the lifespan on physiological and psychobiological variables has been well documented (indicatively, Ford, 2005). Fortunately, the evidence also highlights protective factors for those at-risk or having experienced ACEs, namely safe and nurturing relationships (Crouch et al., 2019) mother-partner relationship, family finances, parent health and wellness, community and neighborhood, and parent child relations (Walsh et al., 2020). Such factors can moderate the adverse effect of ACEs on mental and physical health and are an important factor when looking at promoting health and wellbeing across populations (Hughes et al., 2018).

In this perspective piece, we argue for the protective and therapeutic potential of nature in relation to ACEs. We first present a summary of possible negative effects of ACEs on development and well-being (see also, Purvis et al., 2013). We then highlight through two different literature searches that evidence on the therapeutic and protective potential of nature with ACEs is limited. Finally we present several mechanisms of action through which nature exposure and engagement could act as a significant protective factor with individuals having or at risk of ACEs.

### Physiological Impact of ACEs

Berens et al. (2017) provide a comprehensive overview of disruptions documented among people with ACEs along five physiological axes that link to various impairments. For example, disruptions in brain structure and activity link to executive functioning and emotion regulation impairments; hypothalamic pituitary adrenal (HPA) hyper and hypo activity, and autonomic functioning, link similarly to cardiovascular disease, metabolic dysregulation, and psychopathology.

Ford (2009) explains how neurophysiological changes occur in the brain that experiences adversity (Berens et al., 2017 brain structure and activity axis). In particular, the stress response system of the brain is mobilized under stress conditions –perceived or real threat– to keep all body systems in balance; it does so via the autonomic nervous system. As a result, areas of the brain such as the brainstem and the amygdala within the limbic system are activated to ensure *survival*. Prolonged adversity means irreversible changes in the neurochemical systems too (Perry, 1994). Ultimately disruptions are noted in the neuroendocrine and the immune systems (Oral et al., 2016), for example, the overload of cortisol following activation of the HPA at first, that is then followed by lack of cortisol.

### Psychological Impact of ACEs

The foundations of the neuroanatomical structure of the brain can be located in the genome, however, the experiences have a determining role in the patterns of learning and cognition -and the handling of all new information (Streeck-Fischer and van der Kolk, 2000, p. 908). At the emotional and behavioral sphere, the intermediation of the caregiver is key to emotional development. When caregivers are responsive and empathetic, and experiences positive, emotions (in this instance mostly positive) are experienced first on a physical level; then become verbal and mental (Kooiman et al. (2004). When caregivers are abusive and neglectful or environments emotionally deprived (in ACEs conditions), problems are noted in the area of regulation, spanning to severe forms of affect psychopathology (see for example, Kessler et al., 2010). PTSD is among the most common sequelae from early onset interpersonal trauma (Cloitre et al., 2005) although a linear and straightforward link between the two is questioned (Ehring and Quack, 2010). Forms of major anxiety and have been associated with early onset adversities (Spinhoven et al., 2010). Emotion dysregulation -and depression-in addition provide a pathway from exposure to trauma (emotional abuse especially), to psychopathology in the form of emotional eating (Michopoulos et al., 2015). Similarly, traumatic victimization is associated with oppositional-defiant disorder (after controlling for a host of related variables such as age, gender, family psychopathology, etc.), but this is not the case for exposure to non-victimization trauma (Ford et al., 1999).

In terms of the cognitive functioning difficulties, for example, ADHD and executive functions difficulties have been documented as common problems among children exposed to ACEs and to trauma (for example, Sugarman, 2006). The link, however, between ADHD and trauma seems complicated: the high prevalence of ADHD diagnoses and history of trauma among children, as well as an overlap of symptoms between ADHD and PTSD could be both reasons for misrepresentation of trauma pathology (Conway et al., 2011; Szymanski et al., 2011). Problems in executive functions have also been reported too. While not always so severe to lead to diagnoses of specific disorders, when comparing children with history of trauma to matched controls (see, Bücker et al., 2012) the former perform significantly worse in cognitive areas such as attention, immediate verbal recall, and working memory tests.

### The Positive Effects of Nature and its Therapeutic Applications

Contact with natural environments has been found to have positive physiological and psychological effects on people. Recent large-scale studies and meta-analyses show a stress reduction effect for people immersed in natural environments (e.g., Razani et al., 2019) as well as positive impacts on mental health (e.g., Kotera et al., 2020) and cognitive functions (Zijlema et al., 2017). Moreover, therapeutic nature interventions and ecotherapy have become more widely used both with children and adults, including in the treatment of depression (e.g., Korpela et al., 2016), anxiety (e.g., Nguyen and Brymer, 2018), and ADHD (Kuo and Faber Taylor, 2004). Ecotherapy, adventure therapy, as well as forest school have been used to support optimal development and growth in areas such as self-esteem, resilience and communication for children and young people (e.g., Ward Thompson et al., 2006 and for a review of nature connection intervention in children see Barrable and Booth, 2020). Nature has also been flagged up as a factor for mitigating stress (Ward Thompson et al., 2016) as well as a protective factor for emotional wellbeing (Huynh et al., 2013).

Given the possible overlap between the impact that natural environments have on human physiology and psychology, and the effects of ACEs, we reviewed the literature conducting two separate searches, one for relevant empirical studies that acknowledge nature’s therapeutic potential in relation to ACEs and one for studies that acknowledge nature’s protective potential.

## Search and Results

Inclusion criteria:

1. English language original or translated
2. Both empirical and conceptual articles
3. Published in peer-reviewed journals

We did not include any chronological criteria.

### Search 1: The Therapeutic Potential of Nature in People With ACEs

Search strategy: Major research search engines used, included Scopus, Web of Science and Google Scholar were searched between February and August 2020. The strategy undertaken was:

1. Keywords used included various combinations of the following: “nature,” “natural environment,” “trauma,” “ACEs,” “adverse childhood experiences,” and “therapy” in the title, abstract, or keywords of the article.
2. Titles and abstracts were then scanned for suitability of inclusion in this review. From the articles that were deemed relevant, the authors used a snowballing approach to look through the citing and cited literature.

Results: Nine papers resulted from the searches on the therapeutic potential of nature for children who had experienced ACEs and/or trauma (see Table 1). Two of them offered reviews (Harper, 2017; Summers and Vivian, 2018). Of the remaining seven papers, five directly focused on children or presented at least a case on children and/or youth (Berger and Lahad, 2010; Burgon, 2013; Razani et al., 2019; Berger, 2020; Birch et al., 2020), while five looked at the effects of a nature-based intervention (psychological, educational, or both) on adversity (Berger and Lahad, 2010; Burgon, 2013; Hurly and Walker, 2019; Razani et al., 2019; Birch et al., 2020). Notably, approaches to nature therapy varied much and the same was true for the methodologies employed. Razani et al. (2019) present a group of children screened for ACEs specifically and assessed in different areas (e.g., children’s stress or resilience) at different points in time; Hurly and Walker (2019) used semi-structured interviews and photo-elicitation to explore refugees’ views on the effects of nature-based leisure on their well-being; and Burgon (2013) used ethnographic methods to approach the experience of young people from hard places in a horsemanship program. There were, furthermore, two papers that sought to present nature therapy (Berger and Tiry, 2012; Berger, 2020).

**TABLE 1:** Summary of themes of the reviewed papers on nature’s therapeutic potential.

Type of adversity (or wellbeing aim)	Main themes of nature connectedness approach	Population	Brief description of paper, and/or method, and/or approach to nature related intervention employed	References
Traumatic experience of second Lebanese War by Israeli children.	• Connecting the story of the recovery of the forest from war with work on developing resiliency, advancing flexibility normalizing bad experience and offering safety. • Dramatic distancing (and use of metaphors like children as trees) where children act out their story of their own coping, fears, and connect with inner strengths. • Did not include data on effectiveness of the program.	Young children (in Jewish, Arab, and Druze kindergarten), in northern Israel.	Psycho-educational program in kindergartens.	Berger and Lahad, 2010.
Mental wellbeing and mental-health difficulties.	• Perceived health and wellbeing benefits of nature and nature elements including gaining different sense(s) of self in engaging with the environment; experiencing a sense of escape- nature is what people are not, nature does not judge, it offers a secure sense of self, so it is inclusive; and sense of connection, relationship and care, with trees, houseplants, wildlife or pets; also a theme that nature does not always help. • Effectiveness was measured by participants perception.	Youth (17–27) of which nine had a lived experience of a mental health difficulty (part of the IWUN-improving wellbeing through urban nature study). From diverse ethnic/racial/backgrounds; from deprived areas.	Research/interviews and art workshops on perceived health and wellbeing benefits of nature.	Birch et al., 2020.
Refugee experience/refugee wellbeing.	• Investigated the impacts of nature-based *leisure* on the well-being of refugees. • Participants reported they welcomed the opportunity to connect with others, to learn new activities, involve their families, and as a distraction from their daily lives. • Participants responses supported that nature-based leisure fostered a sense of belonging and refugees’ well-being.	Participants, from three African countries, and Iran (four refugees) in Canada, adults.	Research/semi-structured interviews and photo-elicitation to explore experiences of a 2-day winter camping experience in northern Alberta and how it might foster their well-being.	Hurly and Walker, 2019.
Stress problems/problems in family life; raising awareness of mind-body relationship among students; Israeli children in kindergarten post second Lebanese War.	• Fundamental assumption of Nature Therapy healing can come from reconnection with nature, where people learn to connect with their inner strengths. • It can connect clients to a feeling of inner power and authenticity thus enabling them to develop and express important personal qualities. • Nature therapy bridges elements from Play Therapy, Drama Therapy, narrative approaches, and Gestalt, as well as Ecotherapy, Deep Ecology, Vision Quests, and Adventure and Wilderness Therapies.	Various, adults and groups of Israeli children in kindergarten.	Theoretical paper, drawing from three separate cases to present nature therapy.	Berger, 2020.
Emotional and psychiatric difficulties.	• Links to drama therapy and art therapy. • Exemplification of the how nature’s uncontrollable changes can have a therapeutic power. • How the use of nature can help enter both the fantastic and dramatic realities of participants, which in turn empowers participants and can help mitigate defense mechanisms associated with their difficulties.	Adults.	Application of Nature Therapy, draining from cases to exemplify nature therapy.	Berger and Tiry, 2012.
Children with adversities.	• Three months following a park prescription for nature exposure, each additional park visit per week a child had was associated with a significant improvement in the child’s self-reported resilience. • Clinic and park partnerships be considered as a community asset in addressing toxic stress.	Children 7–17 at a safety-net primary care clinic, low-income families.	Research paper/regression self-reported resilience on part visits, also looking at ACEs, stress, assignment to intervention, and age.	Razani et al., 2019.
Young people experiencing psychosocial difficulties (in foster care/residential facility/youth offending team, also diagnoses for some of them with SEN, autistic spectrum and ADHD).	• Use of Equine-Assisted Learning and Equine-Assisted Therapy (EAL/T). • Reported experience as being calm, (which was very profound for the case with ADHD symptoms), more self-aware- (similarities with exercises in the mindfulness-based stress reduction program), being free, in the moment, authentic, and self-regulated. • Being outdoors provided the right place to be this way.	Young people 11–21 (at risk), participating in a Therapeutic Horsemanship program in United Kingdom.	Research paper-ethnographic approach toward the experience in nature with the horses in the Horsemanship program.	Burgon, 2013.
Child and youth case (not otherwise specified).	• Three thematic areas of practice and research emerged from analysis of included publications: (1) wilderness and adventure therapy, (2) therapeutic camping, and (3) adventure education and physical activity.	63 papers with child populations.	Scoping review paper.	Harper, 2017.
Various physical, mental health, and other disorders.	• Flagging out the different evidence from the literature on the positive effects of time spent outdoors and ecotherapy. • Areas of positive effect included: general medical recovery (e.g., heart rate, blood pressure, surgery recovery, cardiopulmonary rehabilitation), pain reduction, mood and stress (e.g., post-traumatic stress, anxiety, self- esteem, addiction, mental well-being), Attention deficit/hyperactivity disorder, dementia, obesity, other disorders (e.g., vitamin D deficiencies, general mental health issues).	Non-specific.	A review of the literature on ecotherapy.	Summers and Vivian, 2018.

### Search 2. The Protective Potential of Nature

Search strategy: Major research search engines used, including Scopus, Web of Science, and Google Scholar were searched between 20 and 30 October 2020. The strategy undertaken was:

1. Keyword search was conducted using combinations the terms “nature,” “natural environments,” “protective factor,” “adverse childhood experiences,” or “trauma” in the title, abstract, or keywords of the articles.
2. Titles and abstracts were then scanned for suitability of inclusion in this review. From the articles that were deemed relevant, the authors used a snowballing approach to look through the citing and cited literature.

Results: Of the 22 papers resulting from this search two were selected as substantially meeting the criteria (see Table 2). The first presented the positive effects of exposure to 1-day surf training regarded as therapy among youth at risk for intimidation, harassment, discrimination, and bullying-based on identity or status – immigration status, gender-identity/expression, disability, or due to being in foster care or being recipients of the public mental healthcare system (Sarkisian et al., 2020). The second work presented the experience with, and the buffering role of the natural environment among immigrants at risk due to inadequate housing, but also due to “social isolation; language difficulties; underemployment or unemployment; noise pollution; transportation difficulties; and systemic barriers in health, education, and government institutions” (Hordyk et al., 2015, p.76). Results, in both cases show potential.

**TABLE 2:** Summary of themes of the reviewed papers on nature as a protective factor.

Type of risk	Main themes of nature intervention	Population	Brief description of paper, and/or method, and/or approach to nature engagement employed	References
Risk for intimidation, harassment, discrimination, and bullying based on identity or status – immigration status, gender-identity/expression, disability, ethnicity, race, sexual orientation, religion, nationality, or association with a person or group. Youth in foster care and recipients of the public mental healthcare system too.	• One-day surf therapy program (part of the ocean therapy approach). • Use of outdoor water and blue space environments for well-being. • Surfing allows engagement with nature in a symbiotic way, promoting both well-being and self-efficacy. • The 1-day ocean therapy program for youth at-promise includes “(1) an opening talking circle where participants share their experience around a given theme, (2) a surf lesson on land, (3) a surf lesson in the ocean, (4) a second talking circle, (5) a second surf lesson in the ocean, (6) lunch, and (7) a closing talking circle with opportunity to reflect on the theme of the day and other experiences” (p. 6). • 10–12 participants in each session. • The main goal of the program is for participants to be exposed to surfing in an environment that is fun, safe and inclusive.	Youth at-risk (Hispano-Latino primarily, and African American, other ethnicities were only 6% of the group), *N* = 152, ages 6–19.	An observational pre-post-test study, using the Children’s Hope Study (as a measure of a construct linking to resilience) was used to evaluate the program outcomes. The program process was evaluated through rating participant’s drawings.	Sarkisian et al., 2020.
Risk due to inadequate housing but also due to “social isolation; language difficulties; underemployment or unemployment; noise pollution; transportation difficulties; and systemic barriers in health, education and government institutions” (p. 76).	• The study examined the embodied, everyday practices of immigrant children and families drawing from their experiences with urban greenspaces such as parks, fields, backyards, streetscapes, gardens, forests, and rivers. • Structural factors affect social processes in ways that they can give rise to health problems. Adverse social and material living conditions may lead to physiological and psychological stress responses and correspondingly to an increase propensity to health problems and unhealthy coping behaviors. • Positive effects of exposure and engagement with non-threatening forms of urban green spaces on well-being and health (stress reduction theory, attention restoration theory, theory of biophilia). • How familiar sensory stimuli and practices in nature facilitate remembering and belonging.	Immigrants/newcomers in an area with inadequate housing characteristics due to cost, size, and physical conditions. Seven families, 10 adults and 13 children (7–13) comprised the sample.	Interpretative phenomenological analysis of participants lived experience of outdoors and nature, using drawings, the five senses popcorn questions, namely *“what is the first thing that pops into mind when asked to name one thing you have tasted in nature in Canada? Then, one thing you have smelled?*” etc. (p. 77), and semi-structured interviews with the adults (and the children if they wished to join).	Hordyk et al., 2015.

## Discussion

In contrast to the limited evidence on the use of nature specifically with people at-risk or having experienced adversity, ecotherapy, nature engagement, and more specific nature interventions are commonly used therapeutically for –what we regard as– the *effects* of trauma and associated later psychopathology (for the effects see, Felitti et al., 1998, 2019; Burke et al., 2011; Fuller-Thomson et al., 2016; McDonnell and Valentino, 2016; Hughes et al., 2017; van der Kolk, 2017). However, there seems to be a clear gap in research that explores the use of natural engagement in children who are experiencing or have experienced ACEs as a factor that can *mitigate* all or part of the harm or developmental impairment of the child and can act as a protective factor before the harmful impacts of ACEs on development happens. In this sense, in the rest of this perspective paper we wish to bridge this gap by outlining some of the mechanisms of action that may come in useful for practitioners and researchers who are looking to work in this area, a work worth pursuing in our view. As ACEs have notable and varied impact on development, both on the physical and psychological realm, we consider them separately. However, this may be viewed as a false dichotomy, as many of these impacts overlap and are compounded by each other.

### Physiological Areas

Engagement and exposure to natural environments including local green spaces mitigate some of changes and stress responses recounted earlier, as indicated in previous work, mainly in adults (e.g., Ward Thompson et al., 2016). Moreover, studies that have looked specifically at cortisol levels have also found greater access to green spaces to be associated with lower cortisol levels: studies from Japan that have compared salivary diurnal cortisol concentrations in adults who have undertaken forest-bathing versus a control group have also found lower concentrations in the forest-bathing group (Park et al., 2007; Park et al., 2010). Research in urban contexts that has looked at hair cortisol concentrations (HCC) as an indicator of chronic stress found a positive association between living in areas with greater access to natural environments and lower level of HCC (Gidlow et al., 2016). Few studies have looked at children, but a Norwegian prospective longitudinal study in mainstream education found that children (aged 9–10) who experience more outdoor learning, in this case in a forest environment, had a healthier diurnal cortisol rhythm (Dettweiler et al., 2017). Looking at behavioral measures too, Wells and Evans (2003) suggest that nature can act as a buffer for life stresses in children. Overall, although more evidence is required, we believe there is potential for green spaces to be used in order to mitigate some of the physiological effects of trauma.

### Psychological Areas

Natural environments have a positive effect on emotional regulation. A theoretical perspective is given by Richardson (2019) and primarily based on Gilbert’s (2014) three circle model – which in essence simplifies the interaction of physiological and affective factors. Using Gilbert’s model as a starting point, and drawing upon some of the positive affective and physiological experiences that occur in natural environments (including relaxation, feelings of awe and joy) the author proposes that the balance of sympathetic and parasympathetic nervous system responses (as per Porges, 2007) can in fact lead to improved mood and emotional regulation. Earlier theoretical (Johnsen, 2011) and empirical research (Johnsen and Rydstedt, 2013) from Norway supported this potential for using engagement with natural environments as an avenue toward emotional regulation and better psychological health. A recent empirical study from Australia suggests that access to green schoolyards can have a positive impact on self-regulation for young children (Taylor and Butts-Wilmsmeyer, 2020).

Although not directly aiming at specific major anxiety and depression disorders, there is some preliminary evidence to suggest the positive effect of nature and nature-based therapies in these areas too. Maund et al. (2019) worked with adults diagnosed with anxiety and/or depression and ran a pilot on a 6-week wetland nature intervention that showed significant positive effects. In addition, Evans et al. (2003) working with 337 children reported that nearby nature seemed to moderate the effect of stressful events and children’s psychological distress, an effect especially pronounced among children who have experienced high levels of stress. The effect of therapeutic and learning nature-based interventions with children (and adults) with depression, anxiety, but also eating disorders and conduct problems (aggression and defiance) is well-documented in the literature.

Attention Restoration Theory (ART; Kaplan, 1995) puts forward the restorative potential of natural environments, especially in mitigating the effects of stress and directed attention. A systematic review and meta-analysis of the evidence for such a restorative potential, however, has found mixed effects, mostly due to small samples and heterogeneity in tests used and reporting (Ohly et al., 2016). Studies in children, on the other hand, report measurable effects on attention and memory (for example, see Kuo and Faber Taylor, 2004; Ulset et al., 2017). In terms of executive function, studies in both adults (Bourrier et al., 2018) and children (Schutte et al., 2017; Stenfors et al., 2019) have found positive effects of exposure to nature especially as opposed to urban environments. No meta-analytic studies of these exist at the moment, but the effect of exposure to natural environments on human attention, especially in children with pathologies, such as ADHD, merits more research.

## Conclusion

In summary, in this perspective paper we propose that nature holds therapeutic and protective potential for children who have experienced adversity or are at-risk. The brief review of the existing literature indicated that:

1. Children with adversity do present poor physiological and often psychopathological outcomes across the board;
2. Children and adults at-risk or diagnosed with difficulties, physical, emotional, and/or cognitive can benefit from therapeutic nature-based interventions; and
3. To our knowledge there are not specific interventions for children with ACEs or at-risk.

Given the above, it is our view that the mitigating effect of exposure to natural environments needs to be acknowledged, utilized, and researched. In this sense, regular nature engagement can be acknowledged and evaluated for its possible effect as a protective factor for the development of psychopathologies following exposure to ACEs in children. Based on the evidence on mechanisms of action presented earlier, we propose that it is worth investing in designing nature-based programs and developing evidence-based therapeutic and learning interventions targeted specifically to the population of children with risk of being exposed to ACEs.

The approach proposed here (working *a priori* with children at risk or facing adversity rather than working with the effects from ACEs) is seen as granting the positive effects associated with *early intervention* (indicatively, Hester et al., 2003; Evangelou et al., 2007; Lovett et al., 2017): intervening before the appearance of psychopathologies could potentially counter the effects of ACEs altogether. This is especially important as we know that seven of the eight basic categories of adversities are significantly associated with complex adult psychopathology, and pairwise combinations of specific adversities have additive or multiplicative effect in increasing chances for psychopathology across the lifespan (Putnam et al., 2013). For all these reasons, we would welcome the development of nature-based interventions that focus on the above mechanisms of action, expecting that they would optimize children’s chances to escape physical and psychological health challenges in the short-, medium-, and long-term.

## Data Availability Statement

The original contributions presented in the study are included in the article/supplementary material, further inquiries can be directed to the corresponding author.

## Author Contributions

AT was the lead author and conducted the reviews for the manuscripts selected through the searches. AB conducted the literature searches. AT and AB wrote the manuscript collaboratively. Both authors contributed to the article and approved the submitted version.

## Conflict of Interest

The authors declare that the research was conducted in the absence of any commercial or financial relationships that could be construed as a potential conflict of interest.
